# Experimental Demonstration of Sensing Using Hybrid Reconfigurable Intelligent Surfaces †

**DOI:** 10.3390/s25061811

**Published:** 2025-03-14

**Authors:** Idban Alamzadeh, Mohammadreza F. Imani

**Affiliations:** School of Electrical, Computer, and Energy Engineering, Arizona State University, Tempe, AZ 85287, USA; idban.alamzadeh@asu.edu

**Keywords:** metasurface, AoA detection, compressive sensing, reconfigurable intelligent surface

## Abstract

Acquiring information about the surrounding environment is crucial for reconfigurable intelligent surfaces (RISs) to effectively manipulate radio wave propagation. This operation can be fully automated by incorporating an integrated sensing mechanism, leading to a hybrid configuration known as a hybrid reconfigurable intelligent surface (HRIS). Several HRIS geometries have been studied in previous works, with full-wave simulations used to showcase their sensing capabilities. However, these simulated models often fail to address the practical design challenges associated with HRISs. This paper presents an experimental proof-of-concept for an HRIS, focusing on the design considerations that have been neglected in simulations but are vital for experimental validation. The HRIS prototype comprises two types of elements: a conventional element designed for reconfigurable reflection and a hybrid one for sensing and reconfigurable reflection. The metasurface can carry out the required sensing operations by utilizing signals coupled to several hybrid elements. This paper outlines the design considerations necessary to create a practical HRIS configuration that can be fabricated using standard PCB technology. The sensing capabilities of the HRIS are demonstrated experimentally through angle of arrival (AoA) detection. The proposed HRIS has the potential to facilitate smart, autonomous wireless communication networks, wireless power transfer, and sensing systems.

## 1. Introduction

The emergence of reconfigurable intelligent surfaces (RISs) enables the customization of radio channels by reflecting waves in desired directions. Such a reconfigurable reflection capability makes RISs viable candidates for future wireless communication networks [1,2,3,4,5,6,7,8] with improved data rates [9,10,11,12,13,14], increased security [15,16,17], improved backscatter communication [18,19], holographic metasurface communications [20,21], and lower interference [13,15,22,23]. The effectiveness of RISs in directing reflection patterns toward desired locations depends on their ability to access information about these directions. This requires the RIS to have a sensing mechanism that can estimate the angles of incidence and reflection. In wireless communication, the reflection angles can be predicted by measuring the angle of arrival (AoA) of pilot signals. However, this requirement is often overlooked in the current literature on RIS-empowered wireless networks, which typically assumes that this information is readily available. Instead, the focus has been on designing reflecting metasurfaces that can achieve the desired reconfigurable reflection patterns.

Given the vital need to provide information about the dynamic environment to the RIS, recent works have proposed different approaches to addressing this requirement. The primary technique proposed in the literature is the so-called joint channel estimation [24,25]. This approach uses measurements at the transmitter and receiver to estimate the RIS-assisted compound channel. The joint channel is then decomposed to obtain individual channels, i.e., transmitter–RIS and RIS–receiver channels. This approach is computationally demanding, susceptible to noise, and requires a wired or wireless link to provide the necessary information to the RIS.

Several integrated sensing modalities have been proposed in the literature to address the limitations of joint channel estimation. For instance, some works have suggested using a subset of RIS elements to sense the incoming signal [26]. However, these methods fail to retain the full spatial resolution of the sensed data across the RIS surface, as channel information is only available for a limited number of elements, which may limit channel estimation accuracy. Another approach involves using a look-up table for channel estimation, as demonstrated in the ABSense technique [27]. ABSense employs a full-absorption mode, iteratively matching the wave impedance at each element to estimate the electromagnetic properties of the incoming signal via a large look-up table. Although promising, this method is still in development and may require complex protocols for channel sensing, especially in multi-signal environments.

Another approach is to utilize an external receiver to detect signals impinging on a time–space-coded metasurface [28,29]. While this method for allows simultaneous sensing and reflection, it necessitates the deployment of an additional receiver separate from the RIS, adding complexity to the system architecture. Moreover, enabling the temporal modulation of the metasurface’s response further complicates the design. In addition, beam training techniques inspired by the beamforming codebooks recommended for millimeter-wave systems [30] have been proposed to eliminate the need for channel sensing directly at the RIS [31,32]. These techniques rely on deriving adequately diverse information from the codebook during training. However, achieving this requires the effective rank of the codebook to be equal to or close to the total number of RIS elements, resulting in a substantial training overhead, which is particularly problematic in volatile channels.

To address the need to obtain channel information using RISs, a hybrid configuration for RISs has been recently proposed that is capable of both sensing and beamforming operations [33,34,35]. This hybrid RIS (HRIS) utilizes hybrid meta-atoms: elements capable of both reconfigurable reflection and sensing incoming signals. In [36], we used full-wave simulations to present an HRIS capable of sensing incident signals’ AoA using only a few such hybrid elements. To achieve this, we employed the HRIS in an innovative way, enabling it to perform random multiplexing of the incident signal. We proposed a computational processing technique that could analyze this multiplexed signal and retrieve desired information about the incident signal. In this paper, we will present an experimental proof-of-concept of an HRIS that can detect incident AoAs. We will detail the design considerations for this HRIS and illustrate the design space trade-offs made to include necessary DC circuitry that can address HRIS elements and sensing mechanisms on the same layer. For demonstration purposes, we will fabricate a relatively small prototype that will allow us to investigate the experimental AoA sensing capabilities of an HRIS. In [37], we reported preliminary results from this experimental HRIS. This article is a revised and expanded version of [37]. In particular, we will expand on that experimental analysis to include the element design process, investigate the impact of frequency and the number of masks used, and explore intensity-only sensing.

## 2. HRIS Element Design

To design meta-atoms for a practically realizable HRIS prototype, we must balance the trade-off between element size and the space needed for DC biasing and sensing mechanisms. More specifically, we want to be able to connect a coaxial cable to collect the signal coupled to the hybrid element. It is also preferable to develop both DC biasing and sensing waveguides on the same layer to reduce cost and complexity. Considering these requirements, we examined different geometrical parameters to arrive at an HRIS configuration that can be fabricated easily. In this section, we will summarize the results of our analysis in terms of both conventional and hybrid meta-atoms.

### 2.1. Conventional Meta-Atom Design

**Figure 1:**
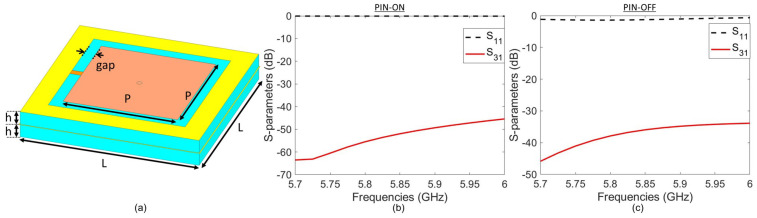
(**a**) Schematic of a conventional meta-atom. In the schematic, the cyan color represents the substrate. All other colors represent copper. (**b**–**c**) The meta-atom’s S-parameter analyses.

We adopted the well-known mushroom structure as the basis of our HRIS design, as seen in [36,38,39,40]. The design frequency of the meta-atom was set at 5.8 GHz. While other meta-atom architectures, such as cELC resonators, can serve the same functional purpose, we proceeded with the mushroom structure, which has been used in previous full-wave simulation studies of HRISs [36,39,40]. We redesigned the mushroom structure for fabrication using an ANSYS HFSS full-wave simulation. The meta-atom consists of two substrate layers, each with a thickness *h*, as shown in Figure 1a. This thickness defines the length of the via of the mushroom, which contributes to the inductive aspect of its resonance. Both layers are assumed to be Rogers RO4003C. The dimensional values of the meta-atom in Figure 1a are listed in Table 1.

A PIN diode connects the central patch of the mushroom structure to the surrounding conductive surface, as shown in Figure 1a. Unlike previous HRIS configurations [36], we used PIN diodes for this design to reduce cost and complexity. Specifically, we used the PIN diode BAR5002VH6327XTSA1 (Infineon Technologies, Hongkong) which was modeled as a capacitor with a 0.15 pF capacitance when *off* and with a resistance of 2Ω when *on*. The PIN diode is controlled by DC signals carried by the via of the mushroom structure. The conducting layer separating the two substrates is the ground for this device. The via passes through this mid-layer and is surrounded by an annular slot.

The incident RF signal couples to the via and the annular slot [41]. This feature can be leveraged for sensing incoming signals by carrying coupled signals towards the sensing circuitry, as was done in [36]. However, this coupling is not enough for sensing at 5.8 GHz. As a result, we used the via only for the DC line and a separate waveguide for sensing, as described later. Nevertheless, the incident signal will always couple to the via, and it is imperative to isolate the RF signal from the DC signal. To address this issue, we attached a radial stub to the via. To accommodate radial stubs (which are usually around a quarter of a guided wavelength) at all the elements without causing significant undesired coupling among them, we used meta-atoms that are one-third of the wavelength, which is slightly larger than the ones used in [33,36].

**Figure 2:**
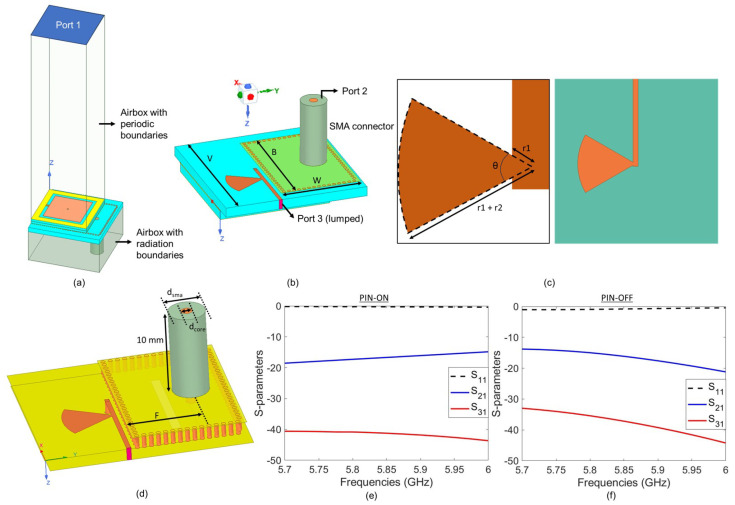
(**a**) Simulation setup and (**b**) the bottom view of the hybrid meta-atom. The substrate is cyan in color. (**c**) The radial stub design (**left**) and the placement of the stub on the bottom surface of the substrate (**right**). (**d**) The placement of the DC biasing circuit, the via cage, and the SMA on the hybrid meta-atom. The SMA is Teflon-filled, with its outer surface coated with copper (not visible in the rendered image). (**e**,**f**) The S-parameter analyses of the hybrid meta-atom.

This meta-atom was surrounded by periodic boundary conditions and excited by port 1, as shown in Figure 2. On the bottom of each meta-atom, we defined a lumped port, namely port 3, which was at the end of the DC microstrip transmission line attached to the stub. The placement of the DC biasing circuitry and port 3 is also denoted in Figure 2. Although the meta-atom in Figure 2 represents our hybrid meta-atom; the DC biasing circuitry and its placement in the conventional meta-atom (Figure 2) are exactly the same as those in the hybrid meta-atom. In this configuration, $S_{11}$ determines the reflection from the element and $S_{31}$ determines the level of RF coupling into the DC transmission line. The results of S-parameter analyses of the finalized conventional meta-atom are provided in Figure 1b,c. Over the frequency range of interest, $S_{11}$ is high while $S_{31}$ is very low—proving the high reflectivity and high DC-RF isolation of this system. In particular, around the design frequency of 5.8 GHz, $S_{11}$ is higher than −2.5 dB, while $S_{31}$ is less than −45 dB when the PIN diode is *on* and less than −33 dB when the PIN diode is *off*.

### 2.2. Hybrid Meta-Atom Design

The hybrid meta-atom is very similar to the conventional one, except we added a rectangular slot to the mid-layer to couple signals into a substrate-integrated waveguide (SIW) for sensing, as discussed in [33,36]. The simulation setup of the hybrid meta-atom comprising the SIW is depicted in Figure 2a. The via walls used to implement the SIW can be seen in Figure 2b. A portion of the incoming signals will travel through the slot in the ground plane and couple to the SIW, which we plan to use to detect the AoA. To do that, we attached an SMA connector to the bottom of each hybrid meta-atom, as shown in Figure 2b. The central conductor of the SMA is extended into the substrate up to the mid-layer. In practice, we can collect the sensed signal by connecting a coaxial cable to this SMA connector. The dimensional details of the SMA and its placement relative to the mid-layer and the via cage are also presented in Figure 2d. Like typical commercial SMAs, we used Teflon as the substrate. The outer layer of the substrate is defined as copper (not shown here). Here, we made the bottom substrate somewhat transparent to allow observation of the rectangular slot (see [36]) in the mid-layer.

We determined the size and dimensions of the SMA connector based on commercially available components. As discussed in [36], we need to balance the reflected signal and the amount coupled to this SMA connector. This trade-off between the signal power in the sensed and the reflected signal, which is governed by the SMA connector’s relative position and the SIW’s size, is vital to our proposed simultaneous sensing and reflection operation. The RIS must be reflective enough to serve its intended purpose practically. On the other hand, the power of the sensed signal must be high enough to estimate the desired parameters reliably. To monitor the level of power coupled to the SMA connector, we placed a wave port at the end of the SMA (see Figure 2b). This port is referred to as port 2. As a result, $S_{21}$ determines the level of coupling. As illustrated in Figure 2e, the $S_{21}$ is above −18 dB around the design frequency when the PIN diode is *on*. It is important to emphasize here that the choice of −18 dB was arbitrary and selected for demonstration purposes, while the level of signal coupling required for sensing is application-specific. One may tune the design parameters of the meta-atom to adjust the coupling level. In addition, the $S_{21}$ is even higher when the PIN diode is *off*, as shown in Figure 2f. Thus, by changing the number of on and off elements in the masks, we can change the power coupling to the shared substrate and the sensing waveguide, as shown by changes in $S_{11}$ and $S_{21}$ in Figure 1 and Figure 2. We can also observe that $S_{11}$ is high for both the *on* and the *off* cases, while also exhibiting a resonance similar to the conventional one near the desired frequency.

Like the conventional version, the hybrid meta-atom is also connected to DC biasing circuitry for independent control, as shown in Figure 2d. This figure captures the challenges in designing the hybrid meta-atom: we want to include the biasing circuitry and the sensing waveguide on the same layer and make them very close to each other. Therefore, the stub size is constrained to almost half of the element size. We chose the stub angle to be $\theta =60^{\circ }$. The dimensional parameters are provided in Figure 2c, while their values are listed in Table 2. A narrow microstrip line is connected to the stub to carry DC signals from the control circuitry to the element. Like the conventional meta-atom, the DC microstrip line terminates in a lumped port (port 3). We can see from Figure 2e and f that the inclusion of the radial stub has resulted in the high isolation of RF and DC.

**Figure 3:**
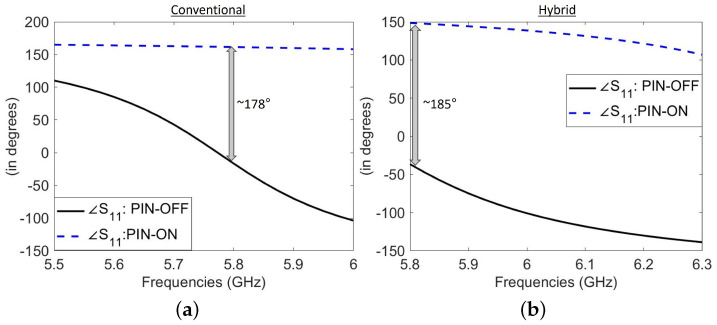
Comparative visualization of the phase of $S_{11}$ between the *on* and *off* states of the PIN diode for (**a**) the conventional meta-atom and (**b**) the hybrid meta-atom.

The ability to effectively change the reflected signal is important when considering an HRIS for wireless communication. In [33,36], we utilized varactor diodes to achieve a continuous phase profile across the HRIS. While theoretically, continuous phase tuning is advantageous for beamforming, DACs are required to realize a continuous voltage across each element. Furthermore, varactor diodes require meticulous characterizations to relate the reflected phase to the associated voltage. To reduce the cost and complexity of this proof-of-concept, which primarily focuses on demonstrating sensing capabilities, we used PIN diodes instead. PIN diodes offer a cost-effective solution, with phase variations of nearly $180^{\circ }$ near resonance between their binary states. As depicted in Figure 3, the phase difference between the two PIN diode states for both types of meta-atom approaches $180^{\circ }$ near the design frequency. This phase difference can be utilized to realize desired beam steering, as shown in many previous studies (e.g., [42,43,44]). Our focus in this article is to demonstrate the capability of this system to be used for sensing purposes.

## 3. PCB Design

With the meta-atom configurations designed, the next step involved their integration into a 2D configuration. In this proof-of-concept demonstration, we opted to showcase their abilities to sense the AoA in the horizontal plane. The results of this study will form the foundation for future work on an HRIS capable of 2D sensing (and beamforming). This choice will simplify the prototype since each column’s diodes will connect to the same DC transmission line.

**Figure 4:**
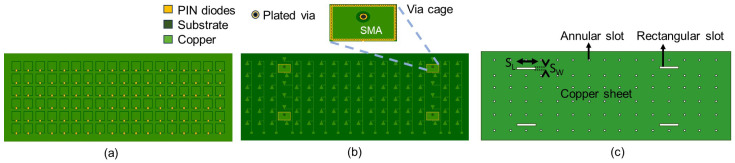
(**a**) Top view, (**b**) bottom view, and (**c**) the mid-layer of the HRIS PCB design.

The 2D HRIS will be rectangular, with an array of 18×6 meta-atoms. In this proof-of-concept demonstration, we used four hybrid meta-atoms, which were grouped into two columns. A top view of this configuration is presented in Figure 4a. In this illustration, PIN diodes are denoted by yellow rectangles. The conductive copper areas on the top surface are colored in a lighter green color. This same color-coding scheme is consistently employed to identify conducting parts across all layers. A darker green color is used to mark the substrate. The substrate beneath the top layer is marked by the thin gap between the patch and the surrounding conductor in Figure 4. Beneath this first substrate layer is the ground plane (or the mid-layer). As shown in Figure 4c, the ground layer contains the same number of annular rings as the number of meta-atoms on the HRIS. In addition, the four rectangular slots of the hybrid meta-atoms are also visible in this figure. The dimensional values of the rectangular slots are given in Table 2.

Four SIWs (via cages) are visible in the bottom view provided in Figure 4b. The central conductor of the SMA will be a blind hole that extends to the mid-layer. A larger circular cut on the conducting bottom surface of the SIW portrays the physical isolation between the SMA central conductor and the conducting plane. The bottom layer also hosts the DC biasing circuitry. The DC biasing circuitry is incorporated into the design to manage and control the operation of the PIN diodes for each column of six elements. As shown in Figure 4b, each via is connected to a radial stub to avoid the RF coupling into the DC circuitry. The rectangular conductive region at each transmission line’s terminus is the designated contact point for routing signals toward the processing and control circuitry.

**Figure 5:**
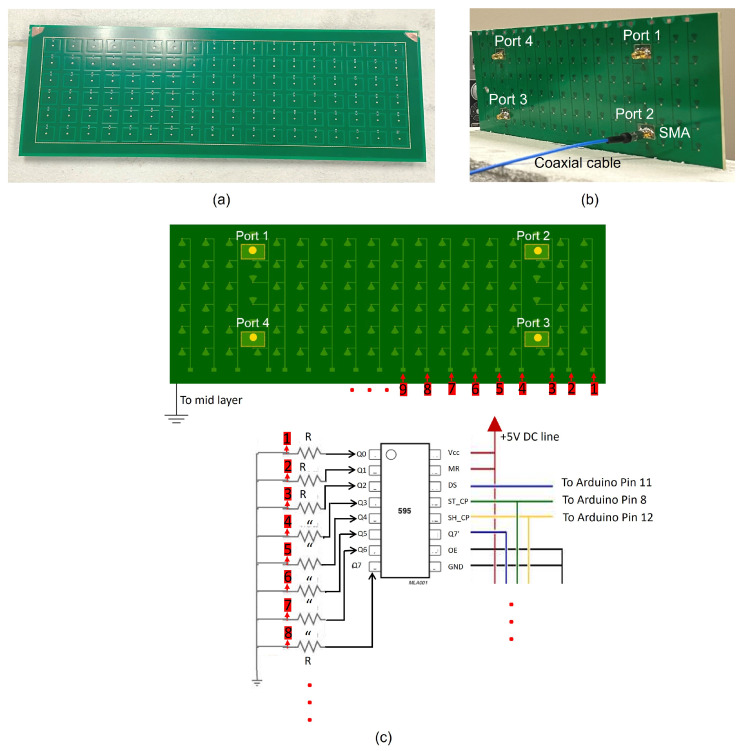
The fabricated 2D prototype of the HRIS. (**a**) Top and (**b**) the bottom views. (**c**) The connection between the control circuitry and the DC biasing circuitry.

As shown in Figure 5a, the top surface of the HRIS is extended equally on all sides by 10 mm. The extended conductor reduces the effect of the edge on the reflective surface. It can also be noticed that the array of elements on the top surface has a rectangular boundary. This boundary consists of vias between the top and the middle layers containing the signal within the mushroom array. This via cage is 315 mm by 105 mm. As shown in Figure 5b, four SMA connectors are soldered to each of the SIWs of the hybrid elements to capture the sensed signals using coaxial cables. One important fact to note here is that in this proof-of-concept demonstration, we used only four hybrid elements and will measure the signal at each separately. This way, we can examine different ways of processing these signals. For example, we can examine the sequential subtraction of the sensed data from all the receiving ports. Or we can subtract column-specific data from one port from those from another. Having access to all four coaxial connectors provides us with these possibilities. The studies presented here could be used in the future to implement specific processes on the HRIS board and connect only one coaxial connector to a sensing unit.

## 4. Control Circuitry

To evaluate the feasibility of the desired sensing operations, autonomous surface reconfiguration is essential to ensure data collection from each incoming signal through the required number of masks without introducing redundant delays. To enable this programmability, we leveraged control circuitry to automate the configuration of elements on the HRIS using a daisy-chained shift register system, as illustrated in Figure 5c. The shift registers are controlled by an ArduinoMega2560 microprocessor (Somerville, MA, USA), which we programmed using MATLAB. The MATLAB script synchronizes the Arduino with other components of the measurement setup to give full autonomy to the measurement process. In essence, the MATLAB code uses this circuitry to send a series of binary sequences to the microprocessor, enabling data collection from multiple masks as the HRIS is illuminated from a given direction. The number of bits in a sequence corresponds to the number of columns on the HRIS, with each output pin of the shift register transmitting a single bit to its respective column of elements. This mapping is illustrated in Figure 5c, where the numbering of output pins aligns with the HRIS columns. As a result, reconfiguration occurs only along the columns for this proof-of-concept prototype, effectively reconfiguring the HRIS surface only along the azimuth. Notably, extending its reconfigurability to two dimensions is straightforward and has been left for future work.

## 5. Measurement Setup

Ideally, we needed to excite the HRIS with a plane wave from known directions to characterize this device’s performance. We also needed to change the masks of the HRIS for each angle and measure the signal from all four coaxial connectors connected to the HRIS. As a compromise, to satisfy all these requirements in our lab setting, we have used a low-gain open-ended waveguide probe affixed to the linear stage as the transmitter. Its radiated field on the HRIS is mostly reflected (or lost due to Ohmic losses). A small portion is coupled to the SIW waveguides behind the hybrid meta-atoms and is captured at the four connectors. By using a linear stage, we can access the reference signal’s exact location (or angular direction). It is worth noting that the HRIS is placed in the far field of the open-ended waveguide probe—which is a more accurate representation of the small antennas used by users in wireless settings. Here, we used a standard WR-137 open-ended waveguide which has dimensions of 34.85 mm by 15.8 mm, resulting in a far field distance of 5.6 cm at 5.8 GHz. However, the HRIS is large enough that the probe is not in its far field for the reference and test measurements. Again, this is closer to many practical scenarios in which RIS-enabled indoor wireless communication networks are used, where users may be in the near field of electrically large RISs.

**Figure 6:**
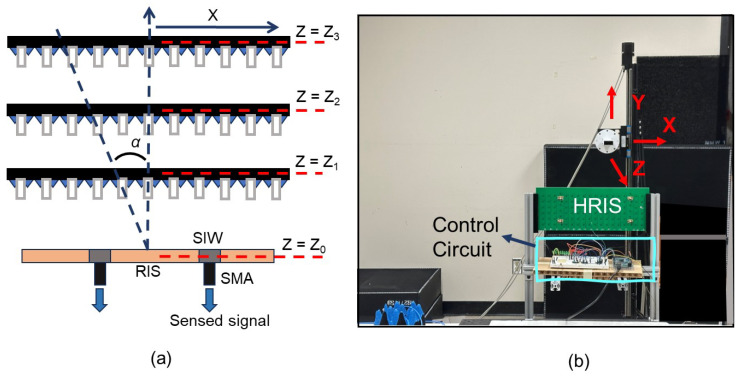
(**a**) The layout of the experimental plan and (**b**) the measurement setup.

The experimental setup is shown in Figure 6. The linear stage was coded to move the probe antenna to an equal distance. At each distance, the HRIS was coded to exhibit the same 40 masks, each containing at least 6 *on* columns. The collection of on and off columns in the masks are selected randomly but are the same in the reference and test measurements. The configuration of the measurement setup is shown in Figure 6a. The probe antenna is connected to one port of a VNA. The coaxial cables attached to the back of the HRIS carry sensed data to a VNA through a mechanical switch. The sensed data are thus captured as $S_{21}$, measured by the VNA. The mechanical switch sequentially allows the VNA to read data from different hybrid elements for every probe location and mask.

### 5.1. AOA Detection

Using the setup described above and shown in Figure 6a, we will collect $S_{21}$ at different distances from the HRIS along the *z* axis (see Figure 6b). The center of the probe is placed at the center of the HRIS, in the *y* axis, for all measurements. At each axial distance, the probe was programmed to move along the *x* axis. The measurement conducted closest to the HRIS, which has the highest SNR and largest field of view, is considered the reference measurement, while the measurements at longer distances will be considered test ones. In this setup, the angular position of the probe moving along the *x* axis is given by the following:

(1) $$\alpha =tan^{-1}\left(\frac{x}{z}\right)$$

During the measurement at all distances, the probe is moved at a periodicity of 30 mm for −480 mm < *x* < 480 mm, with x=0 set to be the middle point of the HRIS. Thus, there were 33 total illumination points during each linear scan. The separation between the adjacent probe locations is about half a wavelength. Adopting a smaller distance between measurement locations may unnecessarily increase the measurement time without increasing the resolution (which is set according to the size of the HRIS). While the sampling resolution along the line-of-scan, $x_{res}=30$ mm, remained the same for different measurement distances, the angular resolution $\alpha_{res}$ is a function of *x* and *z*:

(2) $$\alpha_{res}\left(x,z\right)=\left|\tan^{-1}\left(\frac{z}{x+x_{res}}\right)-\tan^{-1}\left(\frac{z}{x}\right)\right|.$$

Therefore, $\alpha_{res}$ decreases as the scanning distance increases, resulting in denser sampling. On the other hand, the angular span of the scanning is also dependent on the radial distance *z* and it is calculated based on the maximum scan length, $x_{max}=480$ mm:

(3) $$\alpha_{span}=2tan^{-1}\left(\frac{z}{x_{max}}\right)$$

It is worth noting that the AoA for each sample location is, in fact, different depending on the distance of the probe along the *z* axis. In other words, almost all the test AoAs are not in the reference AoAs. The reference distance is set to be 500 mm. Using the measurements at this distance, a reference matrix H is populated. The measurement for a given test AoA at a different *z* distance is given by g. We thus can form the same computational problem as in [36]. This estimation problem is presented as follows:

(4) g=Hf

where f is the parameter to be estimated, which is a vector comprising the same number of elements as the number of sampling points, i.e., 33. The *i*th element of f is 1 if the AoA corresponds to that sampling location and 0 otherwise. We should note that the sensing matrix is not square. Thus we solve this equation using the linear least squares solver, *CGS*, a built-in function in MATLAB 2020a.

### 5.2. Data Processing

The available data from the four sensing points of the HRIS can be combined in different ways. Each combination method will result in a distinct way of defining H and g. To better illustrate these methods, a number is assigned to each of the SMA ports of the HRIS, as shown in Figure 5b. These numbers will also be used to designate the data collected from each port. For example, the data from port 1 are $g_{1}$. Similarly, we can imagine a sensing matrix for each of the ports, i.e., $H_{1}$, $H_{2}$, $H_{3}$, and $H_{4}$. It is crucial to emphasize here that each of $g_{1-4}$ is a m×1 vector and each of $H_{1-4}$ is a m×N matrix. Here, m=40 is the number of masks used for each direction and *N* refers to the number of reference probe locations, which is 33. It is crucial to remember that *M*, the total number of measurements, depends on the number of masks m=40 and how the sensed data are combined. In the following, three different methods for combining the data from these SMA connectors are defined.

1. Method 1: We find the difference between the top and bottom ports, resulting in M=80.
  (5) $$g_{M\times 1}=\left[\begin{array}{c} g_{1}-g_{2}\\ g_{4}-g_{3} \end{array}\right]$$
  (6) $$H_{M\times N}=\left[\begin{array}{c} H_{1}-H_{2}\\ H_{4}-H_{3} \end{array}\right]$$
2. Method 2: We find the difference between the data from adjacent ports, resulting in M=120.
  (7) $$g_{M\times 1}=\left[\begin{array}{c} g_{1}-g_{2}\\ g_{2}-g_{3}\\ g_{3}-g_{4} \end{array}\right]$$
  (8) $$H_{M\times N}=\left[\begin{array}{c} H_{1}-H_{2}\\ H_{2}-H_{3}\\ H_{3}-H_{4} \end{array}\right]$$
3. Method 3: We find the difference between the measured data from a reference port and data from all other ones, resulting in M=120. We have selected port 1 as the reference.
  (9) $$g_{M\times 1}=\left[\begin{array}{c} g_{2}-g_{1}\\ g_{3}-g_{1}\\ g_{4}-g_{1} \end{array}\right]$$
  (10) $$H_{M\times N}=\left[\begin{array}{c} H_{2}-H_{1}\\ H_{3}-H_{1}\\ H_{4}-H_{1} \end{array}\right]$$

### 5.3. AoA Detection Results

For AoA detection, we used the methods defined above to combine the measured data and then computationally solve (4) to find the estimated f, which we denoted as $f_{est}$. Ideally, the $f_{est}$ should be 1 only at the angle of incidence and 0 otherwise. In reality, however, the measured data are disturbed by noise. The reference AoAs are also different from the test angles. Therefore, the values of $|f_{est}|$ are between 0 and 1. We expect to see the maximum of $|f_{\mathrm{est}}|$ to coincide with the reference angle that is closest to the actual AoA [36,39].

**Figure 7:**
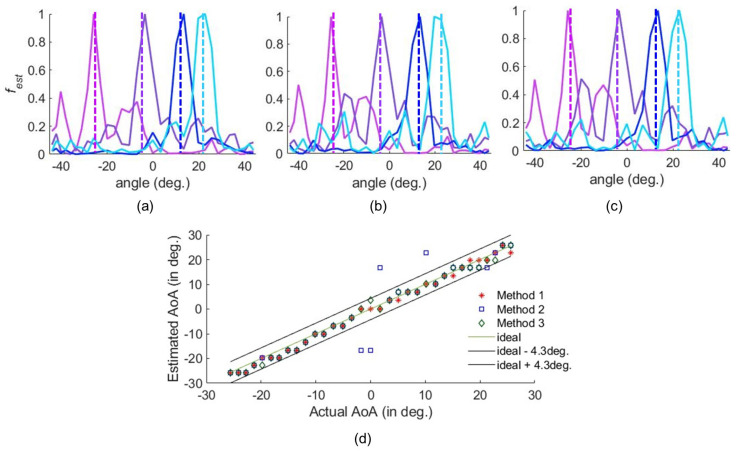
The detection and estimation of the $-24^{\circ }$, $-3.4^{\circ }$, $13.5^{\circ }$, and $22.7^{\circ }$ AoAs at 5.79 GHz when the target is Z=1 m away. The peak detected using (**a**) Method 1, (**b**) Method 2, and (**c**) Method 3. (**d**) The estimation of a wide range of AoAs.

The AoA detection performed under practical circumstances is illustrated in Figure 7, where the $|f_{est}|$ for a number of incoming signals are derived using different methods. The peaks of $|f_{est}|$ corresponding to the AoAs are clearly distinguishable. In Figure 7, the dashed lines correspond to the actual AoAs, which deviate slightly from the estimated AoAs. This is expected since the test AoAs are slightly different from the reference AoA. As a result, there may be an error when estimating the AoA. We define the error tolerance based on the horizontal half-power beamwidth (HPBW) of the HRIS, which is $8.6^{\circ }$. Accordingly, an estimation within $\pm 4.3^{\circ }$ of the actual AoA is considered accurate. The estimated AoA versus the actual AoA is shown in Figure 7d. Evidently, we can detect all AoAs using the fabricated HRIS.

**Figure 8:**
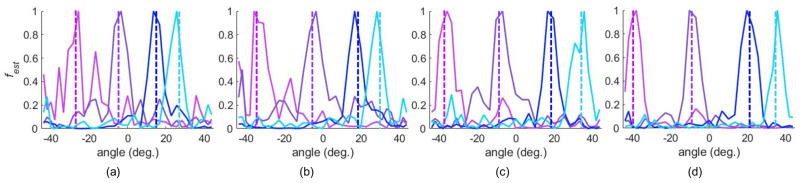
The detection of different AoAs using Method 1 at (**a**) Z=852 mm, (**b**) Z=770 mm, (**c**) Z=661 mm, and (**d**) Z=580 mm. The selected AoAs are denoted using dashed lines.

**Figure 9:**
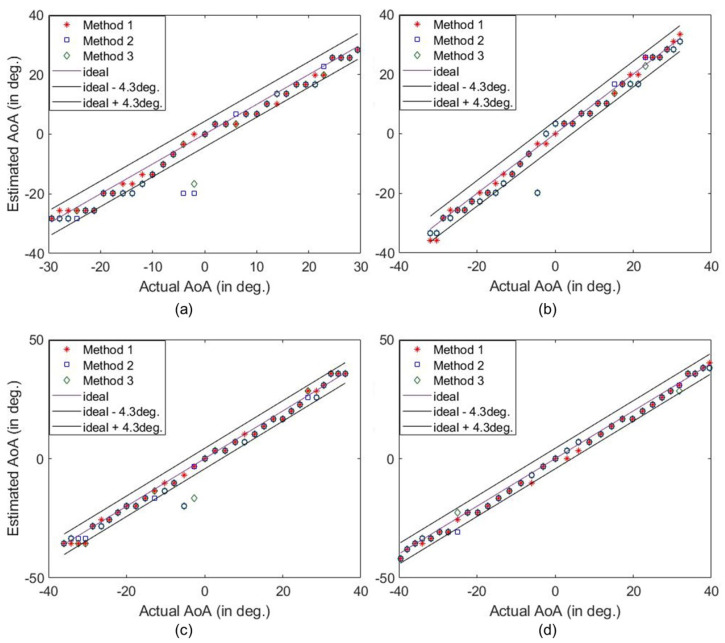
The estimation of a wide range of AoAs at (**a**) Z=852 mm, (**b**) Z=770 mm, (**c**) Z=661 mm, and (**d**) Z=580 mm distances (all detections at 5.79 GHz).

As presented in Figure 8a–d, the peaks corresponding to AoAs are more distinguishable when the source is closer, and thus the SNR is higher. However, the corresponding peaks distinctively dominate the redundant peaks for all different radial distances of the measurement locations. The detection of the AoA at varying distances from the HRIS demonstrates that changing the magnitude and phase of the test signals from that of the reference signal does not impact the sensing performance of the HRIS. The difference in the phase reference plan usually impacts conventional sensing systems when using a finite bandwidth to detect the AoA. However, the proposed sensing mechanism is not dependent on the phase reference because the reference and test measurement are conducted at the same frequency. The relative distance between the reference and the test measurement causes additional phase accumulation, which results in the multiplication of a constant complex number with all measurements. Since we only use the magnitude of $|f_{est}|$, it does not impact AoA detection. A similar performance was also observed in single-frequency imaging using dynamic metasurface antennas, where their performance was not dependent on misalignment or the presence of unknown material [45]. In Figure 9, we plotted the estimated AoA vs. actual AoA at different radial distances for a large set of AoAs. Likewise, an accurate trend in AoA estimation is consistently noted at different radial distances.

Examining Figure 8 and Figure 9, we notice that as the distance increases, the accuracy of the AoA detection decreases. This is expected as the increase in the distance has two impacts: (1) it results in a reduced SNR as the source is farther away and (2) the angular resolution and span of the signal decrease (see Equations (2) and (3)). That means that sources close to each other in the cross-range may not be separable. That is why we can see that the detected AoAs tend to group with each other as the range, *z*, increases. The impact of a low SNR and lower resolutions on the computation solution of (4) is not straightforward, and thus we see an overall trend but with fluctuations. This overall trend, as well as its fluctuations, is noted in Figure 8 and Figure 9. Given the results of this proof-of-concept demonstration, we can conclude that we may need to improve the system’s sensitivity in detecting the AoA in applications involving more considerable distances. This can be achieved by increasing the aperture size, using more masks, and, if needed, using elements with higher coupling or a larger $S_{21}$.

**Figure 10:**
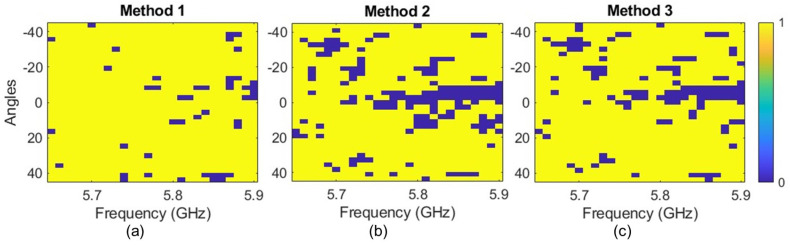
The estimation of a wide range of AoAs at a Z=1 m distance for different frequencies using (**a**) Method 1, (**b**) Method 2, and (**c**) Method 3.

**Figure 11:**
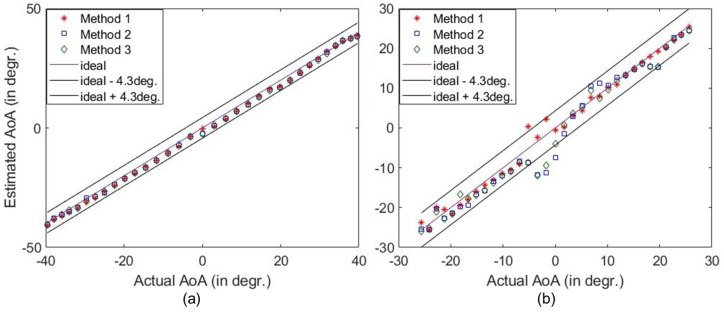
Frequency-averaged estimated vs. actual AoA plot, using different methods, at (**a**) Z=580 mm and (**b**) Z=1 m.

While the proposed HRIS can operate at a single frequency, it is still possible to utilize measurements over a finite bandwidth to increase our accuracy. To show this, we have used data from 26 frequency points between 5.65 GHz and 5.9 GHz. The results of this study are shown in Figure 10 for the three methods described earlier. In this figure, 0 represents inaccurate detection, while 1 denotes accurate detection. The plots in Figure 10 validate the feasibility of AoA detection at multiple frequencies. One way to utilize the bandwidth is to average the detected AoA over multiple frequency points. The results of this averaging are plotted in Figure 11. Evidently, the AoA estimation performance is improved by averaging the estimations from different frequencies.

Examining the results presented in Figure 9 and Figure 10, we can conclude that the method used to combine the data from different hybrid meta-atoms has a noticeable impact on the overall performance of the system. For example, the inaccurate detections seen with Methods 2 and 3 are much higher than those of Method 1 in Figure 10. In the rest of this paper, we will only use Method 1 to process the data. Given the random multiplexing and application of computational processing, it is unclear how each method may perform over others. Our thinking is that Methods 2 and 3 utilize the differences between hybrid elements in the same column, which does not provide much information about the incident signal’s AoA along the horizontal plane. In other words, it adds redundant noisy data that reduce detection fidelity. However, this hypothesis and the best way to combine data need to be tested in a statistical analysis using controlled full-wave simulations in future work. This also indicates that there is a possibility of finding a more optimal method for combining the data, which might require case-specific investigation.

**Figure 12:**
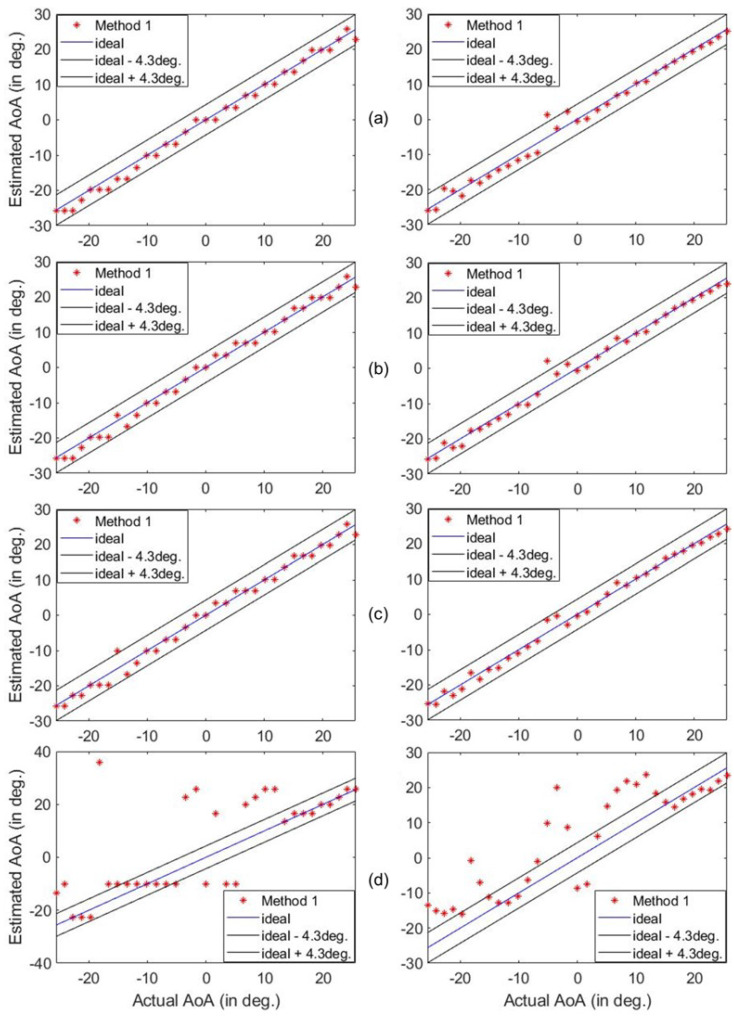
Estimated vs. actual AoA from Z=1 m away when using 32, 16, 8, and 2 masks, as shown in (**a**–**d**), respectively. The estimations on the left side are at 5.79 GHz and the estimations on the right side are frequency-averaged.

Next, we examined the impact of the number of masks on the accuracy of our estimations. To do that, the number of masks was gradually decreased from 40 to 2 masks. This trend is shown in Figure 12. For M=8 (or 4 masks), one AoA is outside the range of acceptable angles. Therefore, the estimation performance of the system saturates at 4 masks. In particular, the estimation accuracy degrades significantly if we reduce the number of masks or measurements further.

**Figure 13:**
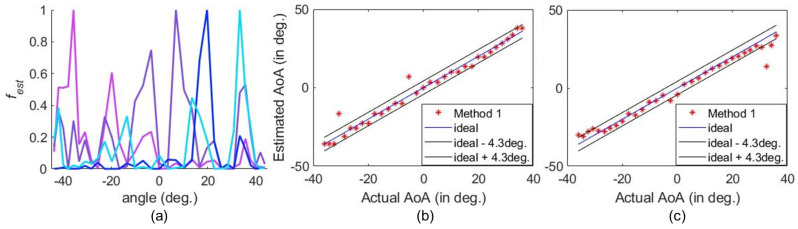
(**a**) Intensity-only estimation of $|f_{est}|$ at z=661 mm. Estimated vs. actual AoA at (**b**) 5.79 GHz and (**c**) after applying frequency averaging.

In the final step, we explore the possibility of detecting the AoA using only the intensity of the data (or received signal strength). Intensity-only AoA detection can significantly reduce hardware complexity [39,40]. To achieve this, we took the magnitudes of the reference sensing matrix H and the measured signal g. The results of this demonstration are presented in Figure 13. As intensity-only estimation disregards half of the complex data—the phase of the incoming signal—it is more susceptible to noise, which is visible in this figure: the redundant peaks are not small, and a successful detection is not possible at larger distances. One can improve this performance by using more masks, as shown in previous works [39,40,46]. The results of Figure 13 verify the efficacy of using the intensity data collected by the HRIS for detecting the AoA.

## 6. Discussion and Conclusions

In this paper, we developed an HRIS that incorporates two types of metamaterial elements: one meta-atom is designed to reflect signals, while the other simultaneously reflects the signal and couples a small portion of it to a waveguide for sensing purposes. We outlined the design considerations for both the conventional and hybrid designs, paying particular attention to fabrication constraints. We fabricated a practical HRIS and experimentally demonstrated its ability to detect the AoA, validating the proposed design and operation. Additionally, we showed that utilizing different frequency points can improve the accuracy of AoA detection. It was confirmed that by leveraging the multiplexing nature of HRISs, we can detect the AoA using intensity-only data.

In this study, we demonstrated that it is possible to detect the AoA using only four hybrid elements. An intriguing avenue for future research involves optimizing the number of hybrid elements, their spacing, and the level of coupling. By employing elements with greater coupling, we can enhance the SNR and potentially reduce the number of hybrid elements required. Conversely, excessive coupling may lead to decreased effective reflection from the structure. These considerations depend on the specific application—whether indoor or outdoor—and must be carefully balanced according to the particular application.

It is worth mentioning that two of the central columns of the HRIS did not exhibit significant changes in their responses due to fabrication issues. These fabrication issues stemmed from poor electrical connections at a few PIN diodes in these columns. Given this issue and the small sample size, we could not investigate the beamforming capabilities of these surfaces. The findings from this study provide a foundation for the future development of a practical HRIS that can sense the environment and change its reflection patterns, enabling a truly autonomous and smart wireless network.

## Figures and Tables

**Table 1:** Design parameters of the meta-atom depicted in Figure 1.

Parameters	Value
Design wavelength	51.7 mm
L	17.5 mm
P	11.2 mm
h	1.52 mm
gap	1.1 mm

**Table 2:** Design parameters for the hybrid meta-atom depicted in Figure 2.

Parameters	Value
V	22.5 mm
B	18.3 mm
W	12.65 mm
F	6.35 mm
$d_{sma}$	4.1 mm
$d_{core}$	1.27 mm
θ	$60^{\circ }$
r1	0.25 mm
r2	5.64 mm
$S_{L}$	11.35 mm
$S_{W}$	1 mm

## Data Availability

The original contributions presented in this study are included in the article. Further inquiries can be directed to the corresponding author.

## Abbreviations

The following abbreviations are used in this manuscript:

RIS	Reconfigurable intelligent surfaces
HRIS	Hybrid reconfigurable intelligent surfaces
AoA	Angle of arrival
cELC	Complementary electric LC (loop-capacitor)
SIW	Substrate integrated waveguide
VNA	Vector network analyzer
PCB	Printed circuit board
CGS	Conjugate gradient squared
